# The Research on Patient Satisfaction with Remote Healthcare Prior to and during the COVID-19 Pandemic

**DOI:** 10.3390/ijerph18105338

**Published:** 2021-05-17

**Authors:** Liliana Hawrysz, Grażyna Gierszewska, Agnieszka Bitkowska

**Affiliations:** Faculty of Management, Warsaw University of Technology, 02-524 Warsava, Poland; grazyna.gierszewska@pw.edu.pl (G.G.); agnieszka.bitkowska@pw.edu.pl (A.B.)

**Keywords:** patient satisfaction, remote healthcare, telehealth services, COVID-19

## Abstract

The issue of research on patient satisfaction with healthcare services took on a completely new dimension due to the COVID-19 pandemic and the developing telehealth services. This results from the fact that during the pandemic, remote healthcare was often the only possible form of care provision to the patient. The COVID-19 pandemic has substantially accelerated the implementation of remote healthcare in healthcare institutions and made it an essential tool for providing healthcare services. The objective of the literature review was to study the research on patient satisfaction with remote healthcare services prior to and during the pandemic. The study featured a literature review of electronic databases, such as: Medline, ProQuest, PubMED, Ebsco, Google Scholar, WoS. The identified empirical papers were classified in two groups concerning the research on patient satisfaction prior to and during the COVID-19 pandemic, and were divided and descriptively synthesised. Certain limitations to the methodical quality of the research were demonstrated as result of the conducted analyses. It was also ascertained that researchers lack clarity on the method of defining and measuring satisfaction prior to and during the COVID-19 pandemic.

## 1. Introduction

The satisfaction of stakeholders, such as patients and service providers, can affect the success and degree of application of telehealth services [1]. The issue of research on patient satisfaction with healthcare services took on a completely new dimension due to the pandemic and the developing telehealth services. This results from the fact that during the pandemic, remote healthcare was often the only possible way of providing care to patients. The COVID-19 pandemic made telehealth services an essential tool for providing healthcare. The application of telehealth services around the world was limited until March 2020. In 2019 in the USA, telehealth appointments constituted 8% of all medical appointments [2]. The primary reasons for scarce utilisation of telehealth services, aside from those deriving from financial issues, included the lack of comfort during the use of telehealth technologies by patients and suppliers. Research conducted in the United States between 2 March and 14 April 2020 demonstrated that the number of telehealth appointments increased by 683% [2]. The level of patient satisfaction with telehealth services was usually very high [3,4,5]. However, the prerequisites for such perception of satisfaction are not entirely clear. First, patients usually highly rated their healthcare in general. Second, the high patient satisfaction with telehealth services could derive from the fact that the appointment was available at a convenient time or due to the time saved because there was no need to drive to the appointment, or that such an appointment could even take place, as was the case during the COVID-19 pandemic [6]. Many studies on satisfaction featured difficulties in identifying the elements of satisfaction desired by the patients and linking satisfaction with a specific service, which in consequence, lowers the information utility of such a study [7]. Satisfaction is deemed important because it can affect the treatment result and the patient’s decision on its continuation [8]. Patient satisfaction is a complex issue and its various aspects can affect telehealth services in a manner that makes the interpretation of results difficult [9]. Furthermore, there are concerns on whether there are methodologies that adequately identify satisfaction and what particular measures represent [10]. Therefore, the paper focuses on the review of studies related to patient satisfaction prior to and during the COVID-19 pandemic. The main objective of the study was to review the research on patient satisfaction with remote healthcare prior to, and during, the COVID-19 pandemic. The conducted analyses focused especially on the methods of defining and measuring patient satisfaction. Due to the fact that the COVID-19 pandemic contributed to a drastic increase in the use of remote healthcare, it was expected that it also entailed greater awareness regarding the methods of defining and measuring patient satisfaction. When comparing the research on patient satisfaction prior to and during the COVID-19 pandemic, it was expected that different operating conditions imposed by the pandemic will also entail a qualitative change in the methods of defining and measuring patient satisfaction. Such a change has been recommended for a long time [11]. The paper is organised as follows. The first part presents a manner of defining and measuring satisfaction provided in literature. The second part features a review of the research on patient satisfaction with remote healthcare prior to the COVID-19 pandemic. The third part features a review of the research on patient satisfaction with remote healthcare during the COVID-19 pandemic. Finally, the fourth part presents the theoretical and empirical implications for patient satisfaction with remote healthcare prior to and during the COVID-19 pandemic.

## 2. Theoretical Background

There is currently much discussion on how to define patient satisfaction in healthcare. In A. Donabedian’s model, patient satisfaction is referred to as the measure of opinions provided by patients [1]. C. Jenkinson et al. pointed out that patient satisfaction seems to mainly reflect the patients’ attitude towards care and the various aspects of this care [11]. In the opinion of M. Tanniru and J. Khuntia, patient satisfaction consists of emotions and perception of the healthcare services provided to them [12]. Other authors specify patient satisfaction as compliance with the patient’s expectations concerning his or her images of perfect healthcare with what is actually provided to him or her, and deem it as an emotional and subjective reaction [13]. Satisfaction is higher, the greater is the compliance with the patient’s earlier expectations, the fewer obstacles in the healthcare system against satisfying own needs and the fewer limitations of rights. Patient satisfaction is a desired result, a measure of quality and the basis for predicting patient behaviours [14,15]. The level of satisfaction is affected by various factors. These factors include, among others, service waiting time, treatment rate, obtaining information on health and the empathy of the medical personnel. The service quality is measured according to subjective criteria. Patients have their own “threshold” of expectations and requirements, their own past experiences and the patient’s feelings and emotions experienced during illness hinder an objective assessment of the situation [4]. It is often stated that patient satisfaction is affected by factors that do not necessarily derive from the treatment process itself, but from his or her expectations towards nurses or doctors and the location of healthcare provision (clinic, emergency department, hospital) [16]. The use of information and communication technologies in service provision contributed to the re-definition of the service provision location and brought healthcare services closer to the patients. Telehealth services are *defined as: “an improvement in the way healthcare provision is conceived and delivered by healthcare providers through the use of information and communication technologies to monitor and improve the wellbeing and health of patients and to empower patients in the management of their health and that of their families”* [17]. In these terms, the research on patient satisfaction is becoming much more important than ever before because in this situation the service provider obtains information about the degree to which its healthcare system satisfies the patients’ expectations. The use of research information allows for adapting the healthcare system to the customer’s needs. Patient satisfaction is commonly studied with the use of questionnaires [18]. M.S. Weaver, J. Lukowski et al. identified 12 questionnaire tools to assess telehealth service interactions [19]. The tools used most often include, among others, the following: Telemedicine Satisfaction Questionnaire (TSQ) [20], Telemedicine Satisfaction and Usefulness Questionnaire (TSUQ) [21], Telemedicine Perception Questionnaire (TMPQ) [22], Patient Experience Questionnaire (PEQ) [23]. The latter part of the paper features a review of studies on patient satisfaction prior to and during the COVID-19 pandemic. The emphasis was especially put on the methods of defining and measuring patient satisfaction prior to and during the pandemic.

## 3. Review of the Research on Patient Satisfaction with Remote Healthcare Prior to the COVID-19 Pandemic

During the time prior to the COVID-19 pandemic, remote healthcare services were developing to a small extent, however their level was high in selected countries, such as the USA, Canada or Australia. The conducted analyses featured a literature review of electronic databases, such as the following: Medline, ProQuest, PubMED, Ebsco, Google Scholar, WoS, with consideration of the patient satisfaction and telehealth keywords. The paper uses a narrative analysis, in the context of a qualitative approach, selecting studies on patient satisfaction with remote medical service provision prior to the COVID-19 pandemic, taking into account primary healthcare, medical specialisations and geographical diversity. The selected studies are presented in Table 1 below. A literature review demonstrated that the provision of remote healthcare services was deemed useful for patients, but there are few complex papers on how patients rated the services and what was the level of their satisfaction with remote appointments. An analysis of the results of the studies on patient satisfaction with remote healthcare service provision prior to the pandemic was rather fragmentary and concerned selected patient groups in selected countries, which featured a various level of development of this type of healthcare services. It mainly concerned selected medical specialisations, the patients’ specific health-related circumstances, and the research samples are not representative and prevent making a comparison. Nevertheless, when compared to outpatient appointments, the patients’ trust towards doctors in remote healthcare is changing and the satisfaction with remote services is high.

Patient satisfaction with remote healthcare services prior to the pandemic is affected by many factors that include, among others, the following:

- criteria related to the service provision and treatment method (medical specialisation): Service availability, first or subsequent appointment-diagnosis, diagnostics, referral for hospitalisation or continuation of treatment,
- behavioural criteria: Building the doctor-patient relations (building communication, trust, perception of the interaction, mindfulness, sufficient time allocated for the appointment),
- systemic criteria: Access to the system, lack of technical problems, electronic medical documentation).

A review of empirical studies points to the lack of uniform research methods and tools used for measuring patient satisfaction with remote services. Various research methods and tools were used, and the number of respondents varied and was difficult to compare.

## 4. Review of the Research on Patient Satisfaction with Remote Healthcare during the COVID-19 Pandemic

The research on patient satisfaction with remote healthcare featured a review of electronic databases, such as the following: PubMED, ProQuest, Ebsco, Scopus, WoS. The analysis covered papers published in the period from 1 January 2020 to 20 January 2021, papers, published and translated into English or German, and included studies that assessed patient satisfaction with telehealth services or telemedicine. The literature review was conducted with the use of a commonly available Internet search engine (Google). Pearling of study reference lists was conducted to identify additional papers. The keywords used in the search were as follows: COVID-19 AND telehealth AND patient satisfaction AND COVID-19; telemedicine AND patient satisfaction AND COVID-19; teleconsultation AND patient satisfaction AND COVID-19. The analyses also featured the consideration of papers published in the period from 1 January 2020 to 20 January 2021, excluding the keyword COVID-19 to prevent a limitation of the results.

The PRISMA guidelines were used during the research. All search results were pooled and the duplicates were removed. Titles and abstracts underwent screening prior to the analysis of full texts to determine their eligibility. All forms of primary research studies were considered in the analyses. Secondary research, such as literature reviews, was excluded but its reference lists were sought after to identify additional studies. Studies were taken into account if they featured measurements of patient satisfaction with telehealth services during the COVID-19 pandemic. Studies were included if the service was provided remotely via audio-visual telehealth platforms, telephone or application. The review was focused on patient satisfaction with telehealth services. All quantitative and/or qualitative measures were taken into account.

Attention was paid to the tools intended for measuring patient satisfaction and the method of communicating with the patient (teleconference, telephone, application) as means for the interpretation of findings and implications for practice and science.

**Figure 1:**
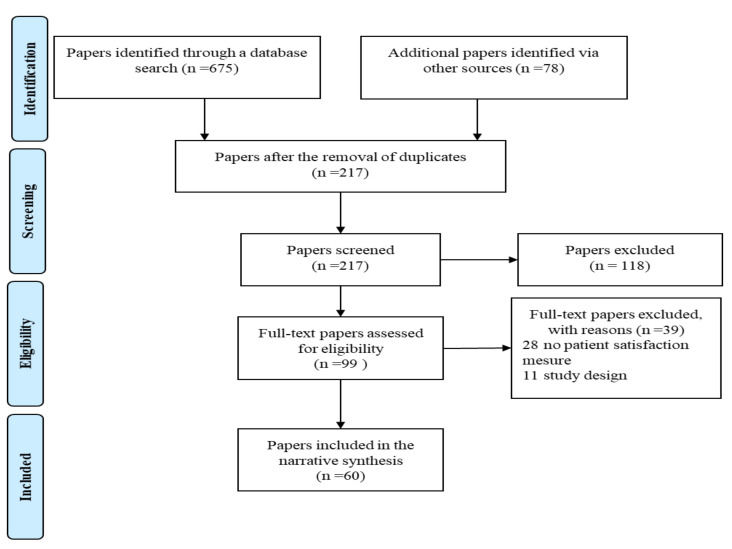
Search strategy and selection of papers.

The initial search featured the identification of 675 studies. After pooling searches and removing duplicates, titles and abstracts were screened, thereby leaving 99 potentially relevant studies. Reference list pearling identified five additional studies. The full texts were retrieved and assessed for eligibility, thereby resulting in the identification of 55 studies eligible for review. The literature selection process is outlined in Figure 1.

### Research Characteristics

The relevant papers were published in the period between 1 January 2020 and 20 January 2021. Half of the studies was conducted in the United States of America. The total number of studies conducted in all European countries is equal to half of the studies conducted in the United States of America [35].

All papers focused on telehealth services featuring remote appointments between patients in their local healthcare centre and a healthcare provider at another centre. 27 papers utilised audio-visual telehealth platforms (i.e., InTouch, Zoom, Doxy.me, Google Hangouts, Apple Facetime, Skype, Upfox, VSee, Epic Warp, Doximity), 17 papers reported using the telephone and 1 study reported using an asynchronous method: Store-and-forward. The communication channel was not specified in two papers, beyond the general statement that it concerned telehealth services.

The clinical areas included surgery (n = 6), otolaryngology (n = 5), remote appointment (n = 5), cardiology (n = 3), neurology (n = 3), oncology (n = 3), orthopaedic (n = 3), rehabilitation (n = 3), urology (n = 3), psychiatry (n = 2), dermatology (n = 1), endocrinology (n = 1), gastroenterology (n = 1), gynaecology (n = 1), ambulatory neurosurgery (n = 1), neurosurgery (n = 1), paediatrics (n = 1), proctology (n = 1), rhinology (n = 1), sports medicine (n = 1), orthodontic (n = 1), ophthalmology (n = 1). The clinical areas were not specified in 7 papers.

The patient satisfaction with telehealth services was measured using a range of different methods, such as questionnaires (n = 52) or numerical rating scores (n = 3). The measures used were often developed for each study’s unique setting, thereby, resulting in the measures’ heterogeneity. In three cases, the authors used measures proposed by others authors [6,35,36,37]. In one case, the authors developed measures based on well-defined instruments: General Medical Council (GMC), Patient Questionnaire, Telehealth Satisfaction Scale (TESS), Telehealth Usability Questionnaire (TUQ) and the Telemedicine Satisfaction and Usefulness Questionnaire (TSUQ) [38]. Nine studies utilised well-defined, known, validated and reliable measures. These measures included the following: SQUIRE 2.0 [39], WCM Press Ganey Medical Practice Survey [5], Telehealth Usability Questionnaire (TUQ) [39,40], Patient Satisfaction Questionnaire-18 (PSQ-18) [41,42,43], Teleconsultation Satisfaction Questionnaire (TSQ) [8], Short Assessment of Patient Satisfaction (SAPS) [44], Patient Assessment of Communication during Telemedicine (PACT) [45] (Table 2).

## 5. Recommendations for Preparing Research on Patient Satisfaction with Remote Healthcare

A lack of attention to the definition of “patient satisfaction” in related studies has been observed for many years [90,91,92,93]. The adoption of a suitable definition of patient satisfaction seems to be a necessary basis for the designing of studies and the development of further papers. Logically speaking, the review of concept and theoretical notions should take place prior to the measurement of this construct, while the analysed papers studied patient satisfaction in the reverse order. There were also papers in which satisfaction was not defined at all [43,46,47,48,49,53,56,61,64,65,66,67,72,80]. Taking into account both the survey questionnaires developed by the authors as well as well-defined, known, validated and reliable measures, these treat satisfaction as a multi-dimensional construct. These dimensions only specify certain aspects of patient satisfaction instead of its essence. The dimensions are usually assessed in various contexts and differ in particular studies [94,95,96]. As result, it is not clear whether some dimensions can be context-specific or whether their application is universal.

The studies on patient satisfaction with remote healthcare commonly use questionnaires [18]. Their popularity as a method of measuring this construct is justified, because it has many advantages. Firstly, confidential questions are well suited for capturing the experiences, observations and attitudes of particular persons. Furthermore, the views of patients and doctors can be assessed and compared independently without endangering their mutual relations. Secondly, the studies can utilise the existing and earlier defined scales, thereby enabling their repetition and comparison of results. Thirdly, such studies allow for data collection from large sample sizes at relatively low costs, which can ensure their representative nature. Fourthly, the relevance and reliability of research tools can be assessed using rigorous and transparent validation methods, giving the researcher confidence that they are adapted to the established objectives and ensure a precise measurement [97].

## 6. Discussion and Conclusions

The first step for the designed studies related to measuring patient satisfaction should be the conceptualisation of the term itself. The second step involved the selection of the measurement tool, possibly from among the existing verified instruments instead of developing new ones. The selection of a verified instrument facilitates the comparison of results from various studies and ultimately creates an integrated collection of knowledge on telehealth services. The tool selected for the study should previously undergo a rigorous assessment of its relevance and reliability, factor structure stability and reactivity to changes over time, especially if it is to be used in the long-term. Insufficient attention is paid to the selection of attributes that are measured with the research instruments and the use of novel instruments, or unverified or unreliable instruments can limit interpretation, prevent comparisons and the advancement of knowledge on the analysed notions [98].

The decision-makers should have the ability to easily interpret the assessment results concerning the provision of remote healthcare services to enable their improvement in their respective healthcare institutions. Patient satisfaction was widely studied, but there are still gaps in the understanding of its role, e.g., in the improvement of the quality of healthcare services. These is needed for modelling factors specific to the situations in which services are provided. Similarly to other researchers studying this phenomenon, the emphasis in this paper has been placed on the fact that patient satisfaction with remote healthcare services can be artificially inflated during the COVID-19 pandemic due to the limited access to outpatient appointments.

However, recent studies on telehealth services demonstrated overall patient satisfaction with any method—video, telephone, application—but this does not mean that improvement measures cannot be implemented. Our understanding of the impact of interactive treatment in outpatient conditions on patient satisfaction is limited. Few papers studied patient satisfaction with telehealth services with the use of validated survey questionnaires which take into account various dimensions of patient satisfaction. More detailed questions in the survey questionnaire can help practitioners understand the specific aspects of remote healthcare that are most important for their patients and adequately redesign their measures. The dominant perspective in the studies on patient satisfaction during the COVID-19 pandemic rarely featured the question of whether the patient was sufficiently satisfied to continue to use this form of consulting if F2F (Face to Face) appointments become available. A large portion of the conducted studies is characterised by high bias, because most respondents had no other option than to choose a remote appointment due to the on-going COVID-19 pandemic. Due to the above, the expressed level of satisfaction does not refer to specific services, but to the availability of any form of healthcare in this difficult period.

If it is possible to choose between face to face and remote healthcare services, many patients prefer the former. Therefore, it seems reasonable that more studies are required that are conducted with the use of survey questionnaires covering more detailed dimensions of satisfaction to assess this discrepancy. For example, patients prefer a limited number of appointments, but healthcare continuity is key for them. However, this aspect is rarely surveyed. The number of outpatient appointments will probably become limited in the nearest future due to the on-going COVID-19 pandemic, while deep and more detailed studies on patient satisfaction with telehealth services can provide valuable information to persons with limit access to patients. A verified questionnaire that encompasses many dimensions of patient satisfaction could be used by researchers from various centres, which in consequence, more reliably compare patient satisfaction with outpatient appointments and remote appointments. This would also allow for the identification of existing gaps in the preferences of potential patients.

An analysis and literature review of the studies on patient satisfaction demonstrate certain limitations concerning their methodical quality. The areas of interest include the small sample size and heterogeneity of the method of defining satisfaction. Furthermore, these areas are measured using unverified instruments. Patient satisfaction was generally not the main objective of the reviewed studies, but rather an additional field of data collection.

## 7. Implications for Practice and Research

The indices for patient satisfaction largely determine the feedback on the provision of remote healthcare services. In order to improve the level of patient satisfaction, it is worth focusing on improving the provision of remote services, especially of aspects important to the patients (service recipients). This includes, for example, prevention of future health issues, providing the patient with full and exhaustive information on the treatment, the further diagnostic and treatment procedure, and even on the impact of the patient’s health issue on his or her family or personal life. It is also important to implement the provision of remote services in combination with outpatient appointments (hybrid model). Taking into account the increasingly competitive healthcare market, institution managers should focus on achieving high or perfect patient satisfaction ratings to improve the quality of the provided services. This can be done (even during the pandemic) if the managers are provided by researchers with guidelines on what does this satisfaction depend on and on the dimensions it encompasses.

The current situation with the COVID-19 pandemic favours using remote healthcare consulting. As demonstrated by the conducted analyses of the studies on patient satisfaction, it is required to adopt a suitable methodical approach, prepare the studies and develop a reliable research tool that takes into account the multi-dimensionality of patient satisfaction. This means not only careful measurement preparation, but also pilot examinations and tool validation. This approach will ensure the reliability and effectiveness of the conducted research studies. The correlations between demographic factors, such as age, gender, health condition, education, and patient satisfaction cannot be disregarded in the analyses. Studies conducted in the future should concern a precise preparation of the analysis, in terms of satisfaction measurement through pilot examinations and tool validation, which in turn, will contribute to the reliability and effectiveness of the papers and the ability of using them in a broader scope.

## Figures and Tables

**Table 1:** Selected characteristics of the research on patient satisfaction prior to the Covid-19 pandemic.

Author	Categories of Care	Type of Measures	Telemedicine Tool	Country
Mendez et al. [24].	Primary Care	own measures	videoconferencing	Canada
Yang Y. Zhang X. Lee P. [25]	Neurology: chronic neurological disorders	own measures	unspecified	Chinese medical platform
Davis et al. [26]	Neurology: chronic neurological disorders	own measures	videoconferencing	USA
Baranowski, M. L., Balakrishnan, V., & Chen, S. C. [27]	Dermatology	own measures	unspecified	USA
Burns et al. [28]	Rehabilitation	own measures	videoconferencing	Australia
Polinski et al. [29]	Primary Care	own measures	videoconferencing	USA
Hilty et al. [30]	Psychiatry	own measures	videoconferencing	USA
Saqui et al. [31]	Gastroenterology:	own measures	videoconferencing	Canada
Simpson et al. [32]	Psychotherapy:	own measures	videoconferencing	Scotland
Zilliacus et al. [33]	Oncology (cancer)	own measures	videoconferencing	Australia
Tokuda et al. [34]	Diabetes	own measures	videoconferencing	USA

**Table 2:** Selected characteristics of the research on patient satisfaction during the Covid-19 pandemic.

Author	Categories of Care	Type of Measures	Telemedicine Tool	Country
Mohanty et al. [46]	ambulatory neurosurgery	own measures	unspecified	USA
Gerbutavicius et al. [47]	ophthalmology	own measures	video	Germany
Byrne & Watkinson [48]	orthodontic	own measures	video	UK
Bhuva, Lankford, Patel, & Haddas [49]	rehabilitation	own measures	mixed (audio/video)	USA
Zhu et al. [50]	surgery	own measures	video	USA
Horgan, Alsabbagh, McGoldrick, Bhatia, & Messahel [51]	surgery	own measures	audio	UK
Haxhihamza et al. [43]	psychiatry	PSQ- 18	video	Macedonia
Smrke et al. [52]	oncology	own measures	audio	UK
Pinar et al. [8]	urology	TSQ	video (virtual room)	France
Campennì et al. [53]	proctology	own measures	mixed (audio/video	Italy
Semprino et al. [54]	pediatric	own measures	video (Whatsapp)	Argentina
Kaur, Galloway, & Oyibo [55]	endocrinology	own measures	audio	UK
Park et al. [41]	unspecified	TUQ	audio	Korea
Itamura, Tang, et al. [56]	otolaryngology	own measures	video	USA
Kodama et al. [57]	cardiology	own measures	not specified/monitoring	USA
Liu et al. [58]	outpatient visit	own measures	video	China
Timmers, Janssen, Stohr, Murk, & Berrevoets [59]	unspecified	own measures	audio/monitoring	Netherlands
Kato-Lin & Thelen [60]	unspecified/acute conditions	own measures	video	USA
Fieux et al. [61]	otolaryngology	own measures	video	France
Layfield et al. [40]	otolaryngology	TUQ	video	USA
Yoon et al. [37]	neurosurgery	Hicks et al. [62]	mixed (audio/video	USA
Dhahri, Iqbal, & Pardoe [63]	outpatient visit	own measures	video	USA
Garcia-Huidobro, Rivera, Chang, Bravo, & Capurro [63]	outpatient visit	own measures	video	Chile
Leibar Tamayo et al. [64]	urology	own measures	audio	Spain
Barca et al., [65]	surgery	own measures	mixed (audio/video	Italy
Rizzi, Polachek, Dulas, Strelzow, & Hynes [66]	unspecified	own measures	video	USA
Casares, Wombles, Skinner, Westerveld, & Gireesh [67]	neurology	own measures	video	USA
Tenforde et al. [39]	rehabilitation	SQUIRE 2.0	video	USA
Ramaswamy et al. [5]	unspecified	WCM Press Ganey Medical Practice Survey	video	USA
Morisada et al. [42]	rhinology	PSQ-18	video	USA
Annis et al. [68]	unspecified	own measures	audio/monitoring	USA
Kirby et al. [69]	sports medicine	own measures	video	USA
Satin et al. [36]	surgery	[70]	video	USA
Chang, Jay, Kalpakjian, Andrews, & Smith [71]	rehabilitation	own measures	mixed (audio/video	USA
Banks et al. [72]	neurology	own measures	audio	Ireland
Barkai et al. [73]	outpatient visit	own measures	video	Israel
Gutkin et al. [74]	oncology	own measures	video	USA
Guinart, Marcy, Hauser, Dwyer, & Kane [75]	psychiatry	own measures	mixed (audio/video	USA
Singh et al. [76]	outpatient visit	own measures	audio	India
Motolese et al. [77]	neurology	own measures	audio	Italy
Kumar et al. [6]	orthopedics	[78]	audio	India
Li, Chan, Huang, & Cheng [79]	surgery	own measures	video	China
Ashmawy et al. [80]	surgery	own measures	video	UK
Darr et al. [38]	otolaryngology	The Pediatric Otolaryngology Telemedicine Satisfaction survey (POTSS): GMC, TESS, TUQ, TSUQ	mixed (audio/video	UK
Futterman et al. [44]	gynecology	The Short Assessment of Patient Satisfaction (SAPS)	audio	USA
Isautier et al. [81]	unspecified	own measures	mixed (audio/video	Australia
Kerr et al. [82]	cardiology	own measures	audio	Ireland
Shafi et al. [83]	orthopedics	own measures	video	USA
Sendagorta et al. [84]	dermatology	own measures	asynchronous: store-and-forward	Spain
Itamura, Rimell, et al. [85]	otolaryngology	own measures	video	USA
Singh et al. [45]	cardiology	Patient Assessment of Communication during Telemedicine (PACT)	video	USA
Taxonera et al. [86]	gastroenterology	own measures	audio	Spain
Schuster-Bruce, Middleton, Macpherson, Pearce, & Evans [87]	oncology	own measures	audio	UK
Shiff, Frankel, Oake, Blachman-Braun, & Patel [88]	urology	own measures	audio	Canada
Khan et al. [89]	orthopedic, ophthalmology, and general medicine	own measures	audio	Pakistan

## Data Availability

Not applicable.
